# Supplementary material for: Synthetic lethality prediction in DNA damage repair, chromatin remodeling and the cell cycle using multi-omics data from cell lines and patients
Source: Sci Rep. 2023 Apr 29;13:7049. doi: 10.1038/s41598-023-34161-4 (PMC10148866; doi:10.1038/s41598-023-34161-4)
Supplement: Supplementary file 1 — Supplementary Information 1. [file 41598_2023_34161_MOESM1_ESM.pdf]

Synthetic lethality prediction in DNA damage repair, chromatin remodeling and the cell cycle using multi-omics data from cell lines and patients

Supplementary information

Magda Markowska[1,2], Magdalena A Budzinska[1,3], Anna Coenen-Stass[4], Senbai Kang[1], Ewa Kizling[1], Krzysztof Kolmus[3], Krzysztof Koras[1], Eike Staub[4], and Ewa Szczurek[1,*]

[1] *Faculty of Mathematics, Informatics and Mechanics, University of Warsaw, Stefana Banacha 2, 02-097 Warsaw, Poland*

[2] *Postgraduate School of Molecular Medicine, Medical University of Warsaw, Zwirki i Wigury 61, 02-091 Warsaw, Poland*

[3] *Ardigen, Podole 76, 30-394 Kraków, Poland*

[4] *Translational Medicine, Oncology Bioinformatics, Merck Healthcare KGaA, Frankfurt Strasse 250, 64293 Darmstadt, Germany*

[*] *Correspondence: szczurek@mimuw.edu.pl*

Contents

1 **SPEA (Synthetic Partner Enrichment Analysis) – formal description**

2 **Supplementary Figures**

Figure 1 The p-value histograms illustrating the SPDD tests results. . . . .

Figure 2 The relation between the positive or negative correlation of expression of SL partners and their occurrence in a shared pathway. . . . .

3 **List of Supplementary Tables provided as separate xslx files**

Table 1 List of focus genes . . . .

Table 2 List of druggable genes . . . .

Table 3 Detailed results for the top clinically relevant 683 focus gene pairs . . . .

Table 4 Detailed results for the top clinically relevant 200 focus vs druggable gene pairs

Table 5 The comparison of SLIDE-VIP to alternative approaches . . . .

Table 6 List of all tested gene pairs . . . .

1 SPEA (Synthetic Partner Enrichment Analysis) – formal description

Let's denote a cell line as cl, number of cell lines as n, dependency score - ds, list of cell lines ranked by decreasing dependency score - L, a position on the list - i, set of cell lines with alteration - $C1 = c1_j : 1, 2, ..., n_a$, set of cell lines without alteration - $C0 = c0_j : 1, 2, ..., n - n_a$. Then the ES score is the (weighted) Kolmogorov-Smirnov (K-S) statistic defined as:

$$ES = \sup_{1<i<n} (F_i^{C1} - F_i^{C0})$$

The ES is the largest difference in F which are the (weighted) empirical cumulative distribution functions:

$$F_i^{C1} = \frac{\sum_{t=1}^{i} |ds_t|^p \ \mathbb{1}_{(cl_t \in C1)}}{\sum_{t=1}^{n} |ds_t|^p \ \mathbb{1}_{(cl_t \in C1)}}$$

and

$$F_i^{C0} = \frac{\sum_{t=1}^{i} \ \mathbb{1}_{(cl_t \in C0)}}{n - n_a}$$

We set the exponent parameter $p = 1$ so that in the calculation of ES we weight the gene A altered cell lines by their dependence score normalized by the sum of the dependence scores over all of the cell lines in our subset.

We estimate the statistical significance (nominal p-value) of the ES comparing it with the set of scores ES_{NULL} computed with randomly assigned gene A alteration status and reordered cell line list. We perform this permutation step 200 times, recompute the ES of the gene set for the permuted data and compile all results to generate a null distribution for the ES. The empirical, nominal p-value of the observed ES is then calculated relative to this null distribution.

2 Supplementary Figures

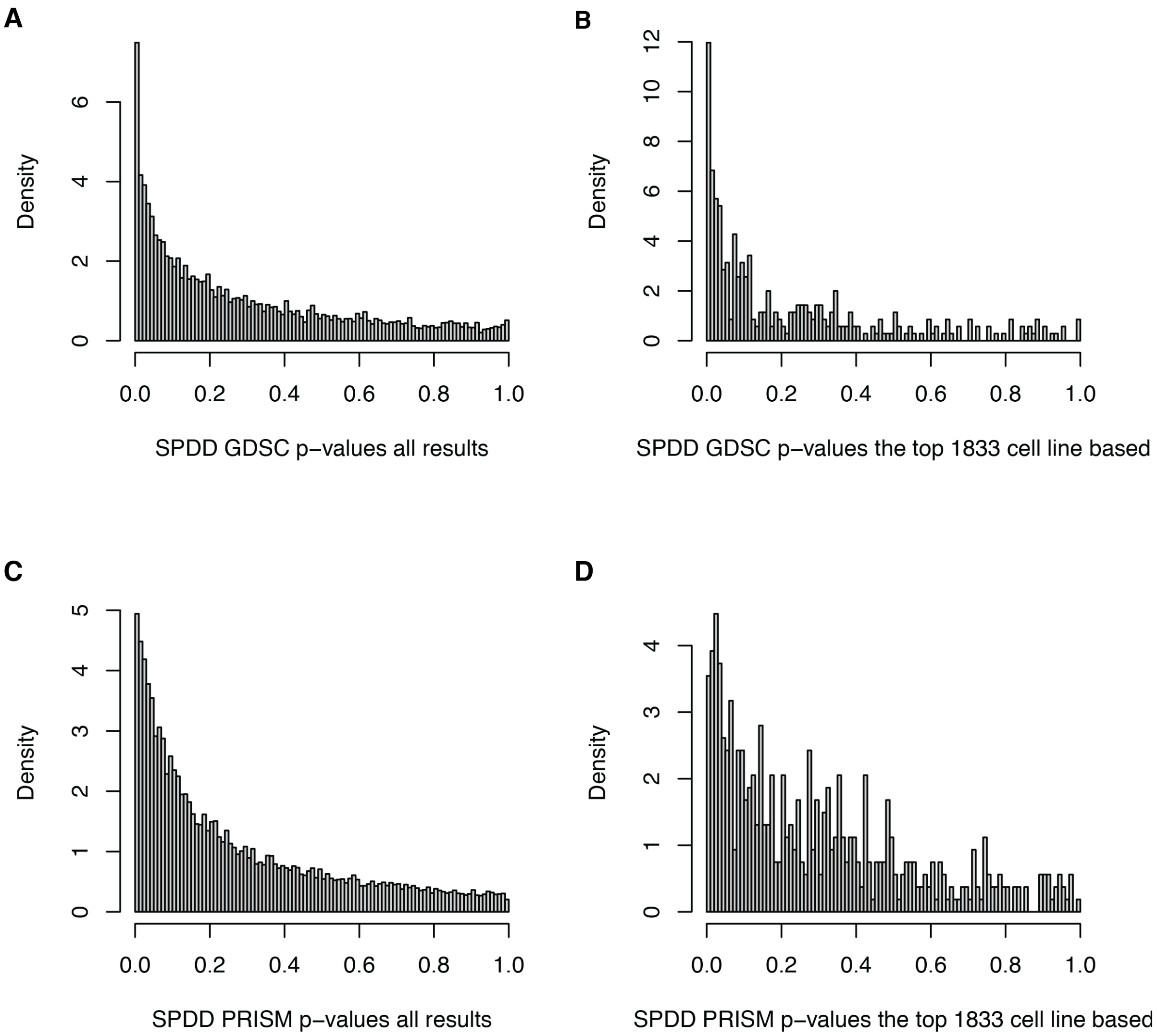

Supplementary Figure 1: The p-value histograms illustrating the SPDD tests results. **A** Histogram of all (26,354) p-values of SPDD test done for GDSC dataset **B** Histogram of 351 p-values of SPDD test done for GDSC dataset (among the top 1,833 cell line based pairs, 351 gene pairs passed the test application criteria for GDSC dataset) **C** Histogram of all (62,026) p-values of SPDD test done for PRISM dataset **D** Histogram of 536 p-values of SPDD test done for PRISM dataset (among the top 1,833 cell line based pairs, 536 gene pairs passed the test application criteria for PRISM dataset)

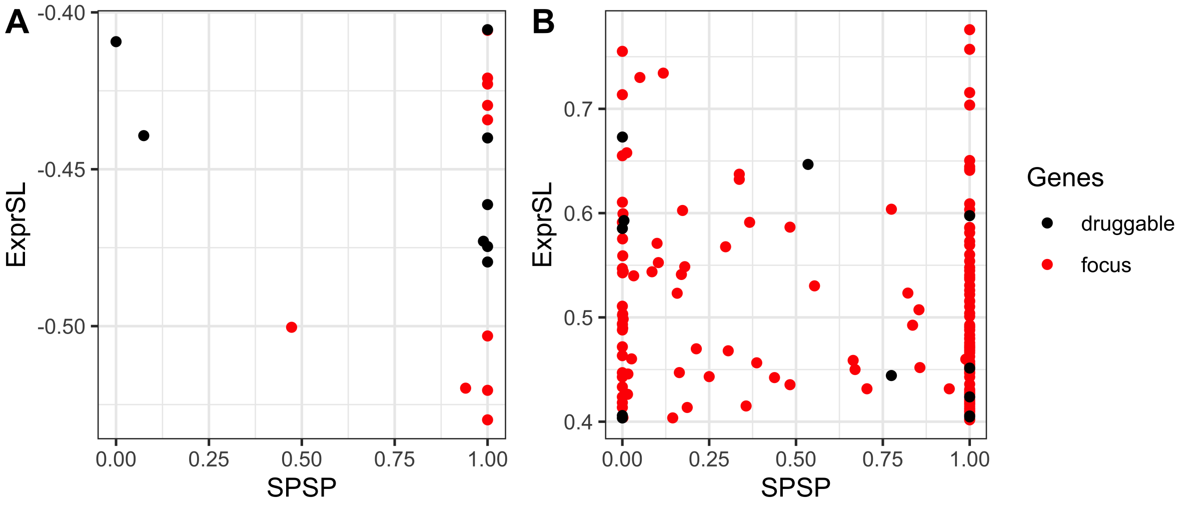

Supplementary Figure 2: The relation between the positive or negative correlation of expression of SL partners and their occurrence in a shared pathway. The plots illustrate the relation between correlation of expression of genes in top SL gene pairs based on ExprSL test (y-axis) and the p-value for the enrichment in common pathways from SPSP test (x-axis). **A** SL pairs with negative expression correlation (Spearman correlation coefficient lower than -0.4 and adjusted p-value less than 0.05) tend not to occur in the shared pathways (SPSP test adjusted p-value more than 0.05). **B** SL pairs with positive expression correlation (Spearman correlation coefficient higher than 0.4 and adjusted p-value less than 0.05) occur in high fraction in the shared pathways (SPSP test adjusted p-value less than 0.05). Focus SL pairs are marked in red and focus vs druggable SL pairs are marked in black.

3 List of Supplementary Tables provided as separate xslx files

Table 1 List of focus genes

Supplementary Table 1 includes a list of all focus genes with an indication of the pathway they belong to.

Table 2 List of druggable genes

Supplementary Table 2 includes a list of all druggable genes with information about the development stage of drugs targeting them.

Table 3 SLIDE-VIP results for the top clinically relevant 683 focus gene pairs

Supplementary Table 3 contains the list of 683 top focus gene pairs sorted according to the final ranking. It also includes details of all SLIDE-VIP tests for those gene pairs.

Table 4 SLIDE-VIP results for the top clinically relevant 200 focus vs druggable gene pairs

Supplementary Table 4 contains the list of 200 top focus vs druggable gene pairs sorted according to the final ranking. It also includes details of all SLIDE-VIP tests for those gene pairs.

Table 5 The comparison of SLIDE-VIP to alternative approaches

Supplementary Table 5 shows the overlap of SLIDE-VIP results with SL experimental screens done by [**Lee2018**]. We treat these experimental results as the gold standard and use them to calculate quality measures for our framework. We do the same for two alternative approaches, namely DAISY [**Jerby2014**] and SL-BioDP [**Deng2019**].

Table 6 List of all tested gene pairs

Supplementary Table 6 includes the list of all (gene A, gene B) pairs that we were able to test with SLIDE-VIP framework. Each pair is presented with test series indication (focus or focus vs druggable) and the SLIDE-VIP SL indication.
